# Effects of *In Utero* Thyroxine Exposure on Murine Cranial Suture Growth

**DOI:** 10.1371/journal.pone.0167805

**Published:** 2016-12-13

**Authors:** R. Nicole Howie, Emily L. Durham, Laurel Black, Grace Bennfors, Trish E. Parsons, Mohammed E. Elsalanty, Jack C. Yu, Seth M. Weinberg, James J. Cray

**Affiliations:** 1 Department of Oral Health Sciences, Medical University of South Carolina, Charleston, South Carolina, United States of America; 2 Center for Craniofacial and Dental Genetics, Department of Oral Biology, School of Dental Medicine, University of Pittsburgh, Pittsburgh, Pennsylvania, United States of America; 3 Departments of Oral Biology, Cellular Biology and Anatomy, Orthopedic Surgery and Oral and Maxillofacial Surgery, Augusta University, Augusta, Georgia, United States of America; 4 Institute for Regenerative and Reparative Medicine, Augusta University, Augusta, Georgia, United States of America; 5 Department of Surgery, Division of Plastic Surgery, Augusta University, Augusta, Georgia, United States of America; Hokkaido Daigaku, JAPAN

## Abstract

Large scale surveillance studies, case studies, as well as cohort studies have identified the influence of thyroid hormones on calvarial growth and development. Surveillance data suggests maternal thyroid disorders (hyperthyroidism, hypothyroidism with pharmacological replacement, and Maternal Graves Disease) are linked to as much as a 2.5 fold increased risk for craniosynostosis. Craniosynostosis is the premature fusion of one or more calvarial growth sites (sutures) prior to the completion of brain expansion. Thyroid hormones maintain proper bone mineral densities by interacting with growth hormone and aiding in the regulation of insulin like growth factors (IGFs). Disruption of this hormonal control of bone physiology may lead to altered bone dynamics thereby increasing the risk for craniosynostosis. In order to elucidate the effect of exogenous thyroxine exposure on cranial suture growth and morphology, wild type C57BL6 mouse litters were exposed to thyroxine *in utero* (control = no treatment; low ~167 ng per day; high ~667 ng per day). Thyroxine exposed mice demonstrated craniofacial dysmorphology (brachycranic). High dose exposed mice showed diminished area of the coronal and widening of the sagittal sutures indicative of premature fusion and compensatory growth. Presence of thyroid receptors was confirmed for the murine cranial suture and markers of proliferation and osteogenesis were increased in sutures from exposed mice. Increased *Htra1* and *Igf1* gene expression were found in sutures from high dose exposed individuals. Pathways related to the HTRA1/IGF axis, specifically Akt and Wnt, demonstrated evidence of increased activity. Overall our data suggest that maternal exogenous thyroxine exposure can drive calvarial growth alterations and altered suture morphology.

## Introduction

Altered craniofacial growth and anomalies often result from a complex combination of genetic susceptibilities, exogenous exposures, and gene/environment interactions [1–8]. Large-scale surveillance studies, case studies, as well as cohort studies have identified thyroid hormones as an important influencing factor in calvarial growth and development; as well as, incidence of craniosynostosis [5, 9–18]. During normal calvarial development, the sutures are formed between the calvarial bones. As the brain grows the connective tissue surrounding the cranial vault expands outward creating tension resulting in stimulation of the osteogenic sutural membranes to produce bone along the osteogenic fronts on either side of the suture area. This results in the enlargement of each bone while maintaining the undifferentiated suture in between [19]. During craniosynostosis one or more of the calvarial growth sites (sutures) fuse obliterating the suture area prior to the completion of brain expansion. The pathogenesis of this disorder is poorly understood but proceeds by bony infiltration within the undifferentiated fibrous tissue of the suture, resulting in synostosis (bony bridging) of the adjacent bones and disruption of normal calvarial expansion [6, 8, 16].

Clinically, the two most commonly affected cranial sutures are the paired coronal sutures, formed between the two frontal and parietal bones, and the sagittal suture, located between the parietal bones [20]. Fusion of the coronal suture results in brachycephaly, impediment of anteroposterior growth of the skull creating a wide, short skull; while fusion of the sagittal suture causes scaphocephaly, or the impediment of lateral growth of the skull while anteroposterior growth continues, producing a narrow elongated skull. Disruption of calvarial growth due to exogenous exposures, such as excessive thyroid hormone, can lead to the loss of sutural growth sites resulting in the inability of the skull to accommodate the rapid growth of the brain leading to serious neurological comorbidities [21–23].

Thyroid hormones are important for normal growth and development of the skeleton in addition to their role in the regulation of metabolism [24–27]. Proper bone mineral densities are maintained by thyroid hormones interacting with factors such as growth hormone and aiding in the regulation of insulin like growth factors (IGFs). In the long bones, thyroid hormone and its receptors target the reserve and proliferating chondrocytes of the epiphyseal growth plates, influencing mineralization and linear growth [28]. Htra1, a serine peptidase, regulates the expression of *Igf1* which acts as a powerful growth factor affecting linear growth, bone cell differentiation, and bone remodeling. In addition, several downstream pathways important for craniofacial development, including Akt and Wnt, are activated or related to IGF1 activity [29–32]. Thyroid hormones have also been shown to be important for skull bone development and for craniofacial growth [33, 34]. Since thyroid hormones act at all stages of bone development and maintenance, it is not surprising that an overabundance of an osteogenic factor such as thyroid hormone has been linked to abnormal growth and development.

Of particular concern for birth defects research is the incidence of maternal thyrotoxicosis, a transient overabundance of circulating thyroid hormone in pregnant mothers. Thyrotoxicosis is estimated to occur in as great as 3% of all pregnancies [35] and results in an exogenous source of thyroid hormone exposed to the fetus, in addition to that which the fetus is already producing by 12 weeks *in utero*. Surveillance data suggests maternal thyroid disorders (hyperthyroidism, hypothyroidism with pharmacological replacement, and Maternal Graves Disease) are linked to as much as a 2.5 fold increased risk for craniosynostosis [5, 36]. At present, the manner in which excess circulating thyroid hormone acts on the developing cranial sutures is not well understood. In this current study, we aim to specifically elucidate the direct effect of *in utero*, exogenous thyroxine exposure on cranial suture growth and morphology in wild type mice. We hypothesize exposure to exogenous thyroid hormone *in utero* will alter craniofacial growth in a dose-dependent manner. Further, we postulate that these induced alterations in growth will involve the downstream Akt and/or Wnt signaling.

## Materials and Methods

### Animal Model in vivo Exposure

Adult, wild type, C57BL6 (*Mus musculus*, Jackson Laboratories, Bar Harbor, ME, USA) male and female mice were utilized to produce *in utero* thyroxine exposed litters. Animals were bred and separated at ~E13 of pregnancy. At this time, previously described dose ranges of levothyroxine (Synthroid), known to increase T3/T4 exposure without causing amelioration of maternal Thyroid Stimulating Hormone levels, (control dose = no treatment; low dose ~ 167 ng per day; high dose ~ 667 ng per day), were added to the drinking water provided to pregnant dams [37–45]. Treatment continued until birth of the litters at ~E20. Mouse pups were grown to 15, 20 and 25 days post-natal (pn) when they were sacrificed via CO_2_ overdose with cervical dislocation utilized as a secondary method and whole skulls were collected and fixed with 4% paraformaldehyde for 48 hours and then switched to 70% Ethanol for micro computed tomography (μCT) analysis. Samples were then further processed for histological investigation. A set of randomly selected skulls were not fixed but were used for RNA (n = 3 control and n = 3 high dose) and protein (n = 3 control and n = 3 high dose) isolation from excised suture tissue. Animal use protocols were approved by Georgia Regents University Institutional Animal Care and Use Committee (2011–0365), and the Medical University of South Carolina Institutional Animal Care and Use Committee (AR#3341). All breeding procedures were carried out in an Association for Assessment and Accreditation of Laboratory Animal Care International accredited facility where all husbandry and related services are provided by the Division of Laboratory Animal Resources. Animals were housed in ventilated racks with automatic water and feeders providing mouse TEKLAD pellets with a 12 hour light-dark cycles. Certified technical personnel and registered veterinary technicians provide daily observation and handling of lab animals. Signs of dehydration and pain as indicated by hunched and lethargic behavior were monitored to assess animal health. All procedures and the reporting thereof are in compliance with the Animal Research: Reporting in Vivo Experiments (ARRIVE) guidelines [46].

### Microcomputed Tomography (μCT) Analysis

μCT images were obtained on 15, 20 and 25 day post-natal mouse pup skulls with a SkyScan 1174 (Kontich, Belgium) at a 22.57 μm voxel resolution. Scans were obtained on 181 animals (Male = 42.8%; Female = 43.3%; Undetermined = 13.9%). Mouse skulls were reconstructed with NRecon v1.6.4.8 (BrukermicroCT, Kontich, Belgium) as previously described and imported into Amira v5.0 where it was exposed to a Gaussian Smoothing image filter (r50.3 in X, Y, and Z dimensions; isometric kernel size53) to reduce extraneous noise in the images [47]. Threshold settings were then set to only visualize bone volume within the skull. Measurements of the length and width of the cranial vault were collected by a single experienced rater (TEP) from each reconstructed mouse skull. Cranial vault length was defined as the linear distance between landmarks opisthion and nasion. Cranial vault width was defined as the linear distance between the left and right sqzy landmarks, which are defined as the point of junction between the posterior zygomatic arch and squama of the temporal bone. The above landmarks can be visualized at the following website: http://getahead.psu.edu/viewer.html?id=Adult_Mouse_Skull. Cranial vault width and length measurements were used to define the cranial vault index (width x 100 / length) to further analyze 3D morphometric alterations due to treatment. Additionally, the widths of the coronal and sagittal sutures were measured at 25, 50, and 75 percent of their length as defined by the distance from the bregma to the pterion and from the bregma to the lambda, respectively. The width of the suture was defined as the distance between bony fronts at each of these points. Measures were compared at each time point by split-plot ANOVA or Kruskal-Wallis where appropriate by postnatal time point or suture for effects by dose where applicable; p≤0.05 was considered significant for post-hoc Bonferonni analyses. Sex of the pups was recorded for future post-hoc investigation but was not considered a factor in current analyses. All statistical analyses were completed using SPSS 23.0 (IBM, Armonk, NY, USA). All measurements are presented as mean ±SEM.

### Hematoxylin and Eosin Suture Histomorphometry and Immunohistochemistry

After μCT scanning, representative samples (n = 3 per group) from the control unexposed and high dose exposed groups were decalcified in 0.25M EDTA at pH 7.4 for 10 days with changes every 3 days. The sutures were isolated from the calvaria, dehydrated in a graded series of ethyl alcohol (70–100%), cleared in xylene, and embedded in paraffin. Prior to embedding, samples were cut in half posterior and parallel to the coronal suture and the front half of the calvaria was again bisected along the sagittal suture to allow for embedding in an orientation that allowed for cutting through both sutures at 8 μm using a rotary microtome prior to mounting on Super Frost Plus (ThermoFisher Scientific, Waltham, MA, USA) glass slides for histology. Hematoxylin and eosin staining proceeded by standard protocol. Three sections at least 30 μm apart per specimen per suture were used for histomorphometric analysis. Stained sections were photographed using a Motic Inverted Microscope with attached camera (Motic, British Columbia, Canada). Standardized variables including total suture area, suture width at the dural, middle, and periosteal edges, and suture height were measured using Image J Software (National Institutes of Health) [48]. Measures were compared for the coronal and sagittal sutures using a split-plot ANOVA to investigate each suture for effects by dose, p≤0.05 was considered significant.

For immunohistochemistry, antigen retrieval was achieved with 10mM Sodium Citrate Buffer or Tris-EDTA Buffer (2 minutes microwave). After cooling, endogenous peroxidase activity was blocked with 3% hydrogen peroxide in methanol (10 minutes) and then sections were washed in phosphate-buffered saline (PBS) and blocked in 1% goat serum with 1% bovine serum albumin (10 minutes). Sections were then incubated with the following primary antibodies for one hour at room temperature or refrigerated overnight: Thyroid Receptor Alpha (AbCam, Cambridge, MA, USA, ab53729, 1:50), Thyroid Receptor Beta (AbCam ab180612, 1:50), PCNA (AbCam ab18197, 1:3000), ALP (AbCam ab108337, 1:250), HTRA1 (AbCam ab38611, 1:50), IGF1 (AbCam ab9572, 1:50), Active Caspase 3 (AbCam ab2302, 1:75). Next, sections were washed with PBS then incubated with horseradish peroxidase conjugated secondary antibody for one hour (AbCam, ab6721). Finally, diaminobenzidine (DAB) (Vector Laboratories, Bulingame, CA, USA) chromagen was used to identify immunoreactive structures and the sections were counterstained with hematoxylin for orientation. At least 3 sections per individual per treatment for each suture (sagittal and coronal), for each target were analyzed using Image J Software and the IHC Profiler Open Source Plugin for automated scoring of immunohistochemical staining [49], where the output comprised of percent positivity. Measures were compared for isolated coronal and sagittal sutures via t tests or Mann Whitney U where appropriate for effects by dose; p≤0.05 was considered significant.

### Western Blots on Suture Tissue

A set of randomly selected 20 day skulls (n = 3 control and n = 3 high dose) were not fixed, but were used for protein isolation from suture tissue. The coronal and sagittal sutures, including the osteogenic bony fronts, were identified and excised using a small periosteal elevator and scissors to extirpate. The extirpated tissue was then homogenized in a liquid nitrogen cooled mortar and protein was extracted with RIPA buffer (ThermoFisher Scientific). Total protein was quantified using a Bradford assay (ThermoFisher Scientific) according to manufacturer protocol. Whole tissue protein extracts were separated by 10% SDS-PAGE. Equal amounts of protein per lane were loaded and transferred onto polyvinylidene difluoride membrane (BioRad, Hercules, CA, USA). The blots were probed with the following antibodies diluted in Tris-buffered saline, 0.1% Tween 20 with 5% (wt/vol) bovine serum albumin: AKT (Cell Signaling, Danvers, MA, USA, 9272; 1:1000), pAKT (Cell Signaling 9271; 1:1000), active Caspase 3 (AbCam ab49822; 1:500), PCNA (AbCam ab18197; 1:250), B-catenin (AbCam ab2365; 1:10,000), GAPDH (AbCam ab181602; 1:10,000) and B-actin (Cell Signaling 4967; 1:10,000). Incubation with horseradish peroxidase-conjugated anti-rabbit IgG (AbCam ab6721; 1:3000) followed. The protein was then visualized by enhanced chemiluminescence ECL Clarity (BioRad) detection reagents. Measures were compared for isolated coronal and sagittal sutures via t-tests or Mann Whitney U where appropriate for effects by dose; p≤0.05 was considered significant.

### Tissue Based Quantitative Polymerase Chain Reaction

A parallel set of randomly selected skulls (n = 3 control and n = 3 high dose) were not fixed, but placed in ice cold RNAlater (ThermoFisher Scientific). Subsequently, the coronal and sagittal sutures were identified and isolated as for the total protein isolation. The extirpated tissue was then homogenized in a liquid nitrogen cooled mortar and digested in TRIZOL (ThermoFisher). RNA was then isolated using the Qiagen RNEasy mini kit (Qiagen, Valenica, CA, USA) according to manufacturer’s protocol. Quantity and quality of RNA was assessed using a Synergy H1 Microplate reader and a Take3 Microvolume Plate (BioTek, Winooski, VT, USA). Complimentary DNA Synthesis was performed using Superscript II Reverse Transcriptase and random hexamer primer following manufacturers protocol (ThermoFisher Scientific) and then subjected to quantitative PCR using Applied Biosystems TaqMan Gene Expression Master Mix and the following targeted TaqMan gene expression assays for the *Igf1* pathway: *Htra1*, *Igf1*; for proliferation; *Ki67*, *Ccnd1*, *Ju*n*;* for apoptosis; *Caspase 3*, *Bax*, *Bcl2*; and for osteogenesis; *Runx2*, *Alp*, and *Bglap*. In addition, targets of the Akt pathway including *Akt1*, *Irs1*, *Mtor*, *Nfkb1*, *Rankl*, *Vegfa*, and *Foxo1*, and the Wnt pathway including *Ctnnb1*, *Dact1*, *Zbed3*, *Lrp5*, *Lrp6*, *Lef1*, *Tcf7*, *Dkk2*, *Dkk3*, *Sfrp1*, *Frzb*, and *Sfrp4* were interrogated. Data were normalized to 18S ribosomal RNA expression by ΔCT. Quantitative data were compared for gene expression change due to treatment by ΔΔCT methodology. We used statistical analyses for qrt-PCR data as previously published to determine statistical differences for gene expression after thyroxine treatment for targets of interest [50]. Statistical analysis was completed using SPSS 23.0. Differences were considered statistically different if p≤0.05. Taqman assay information is available in S1 Table.

## Results

### In utero Thyroxine Exposure Alters Vault Expansion and Cranial Suture Morphology

**Fig 1:**
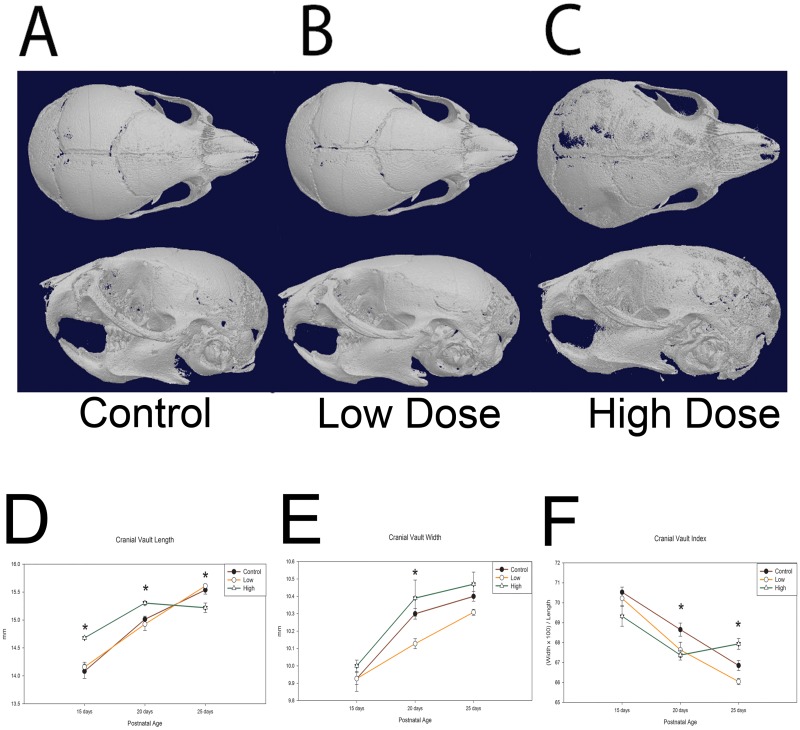
Effects of *in utero* thyroxine exposure on post-natal craniofacial morphology. A-C. Representative 3D μCT skull reconstructions demonstrating dysmorphology in the high dose exposed of 25 day animals (C). D. Cranial vault length measures for control, low and high dose exposed C57BL6 mice at post-natal days 15, 20, and 25 with high dose demonstrating significantly more vault length at 15 (p = 0.046) and 20 days (p = 0.05) than control and low dose, then ceasing to lengthen at 25 days, p = 0.005, having significantly smaller length than both control and low dose respectively (see Table 1 for n). E. Cranial vault width measures for control, low, and high dose exposed mice at post-natal days 15, 20, and 25 with high dose demonstrating significantly more width at 20 days compared to low dose, p = 0.028 (see Table 1 for n). F. Cranial vault index for control, low, and high dose exposed mice at post-natal days 15, 20, and 25 with control exhibiting greater values at 20 days compared to high dose, p = 0.016, and high dose having significantly greater values at 25 days compared to both control (p = 0.005) and low dose (p<0.001). All measurements are presented as mean ±SEM. * p = 0.05–0.011.

Representative μCT reconstructions are included in Fig 1. Note gross dysmorphology observed in the high dose exposed representative compared to control or low dose exposed (Fig 1A–1C). Cranial growth measures were interrogated at three post-natal time points after *in utero* thyroxine exposure. There was approximately equal representation of sex among the 181 resulting samples (Male = 42.8%; Female = 43.3%; Undetermined = 13.9%) (Table 1). Results suggested arrest in growth in cranial vault length dimension after 20 days post-natal for the high dose (~667 ng per day) exposed group with control and low dose (~167 ng per day) having greater length measures at 25 days, p = 0.005 (Fig 1D). High dose appeared to have some compensatory growth in cranial vault width, with significantly greater measure at the 20 day time point compared to low dose exposed, p = 0.028 (Fig 1E). For cranial vault index these differences are accentuated where the control group had the greatest values at 20 days, and was significantly different from the high dose, p = 0.016. At 25 days, the high dose group displayed significantly different values from control and low dose groups, p<0.005 (Fig 1F). Greater index values represent a more brachycranic phenotype suggesting growth trajectory alteration of the coronal suture for the high dose group.

**Table 1:** Group Sample size of C57BL6 pups.

	15 Day Pups	20 Day Pups	25 Day Pups
Control	26	18	23
Low (~167 ng per day)	10	18	32
High (~ 667 ng per day)	6	17	31

**Fig 2:**
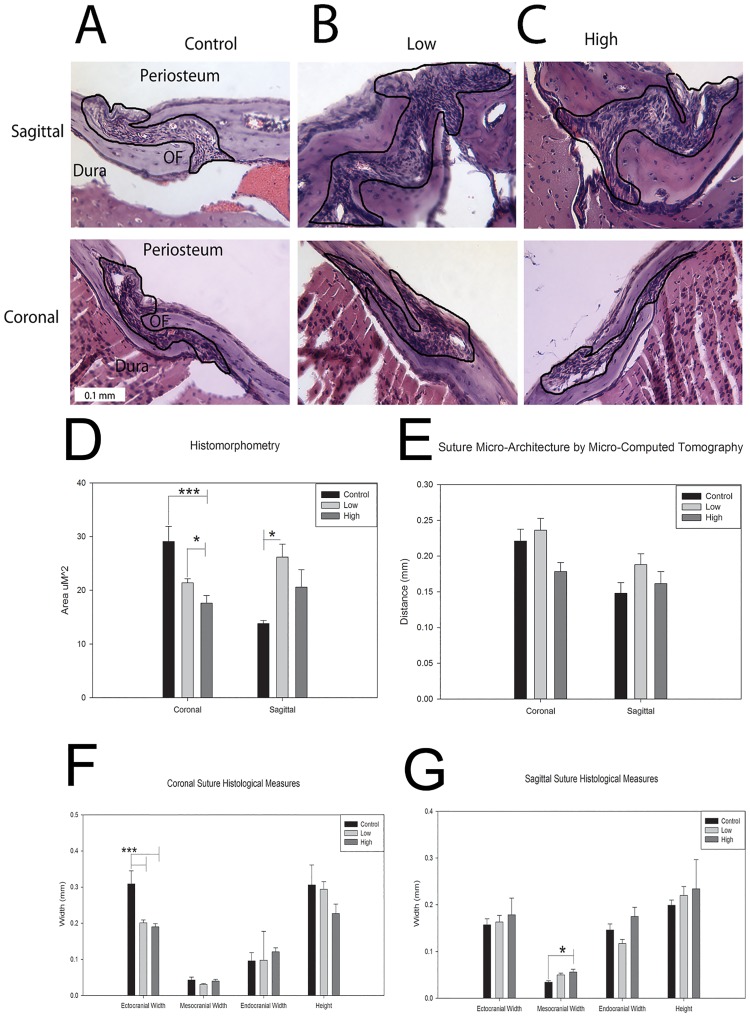
Effects of *in utero* thyroxine exposure on post-natal cranial suture morphology. A-C. Sagittal (top) and coronal (bottom) sutures for control, low and high dose exposed 25 day post-natal mice stained with hematoxylin and eosin. Sutures are outlined with periosteum above, osteogenic fronts (OF) on either side, and dura below. D. Histomorphometric analysis of sagittal and coronal suture area from 25 day post-natal mice revealing a dose dependent significant reduction in coronal suture area for high dose compared to control (p<0.001) and low dose (p = 0.005). A compensatory significant increase in the sagittal suture area of the low dose animals was seen compared to controls (p = 0.027) (n = 3 per exposure). E. μCT assessment of suture micro-architecture analysis suggests diminished coronal suture width for high dose group compared to control and low dose for the suture, (not statistically significant, p = 0.070) while in the sagittal suture, the low dose group had a greater but not statistically significant width than control (n = 9 per exposure). F. Measurements of ectocranial, mesocranial, and endocranial widths for the coronal suture with control having significantly increased width in the ectocranial space compared to both low and high doses (p<0.001 respectively). Measurement of the height dimension showed a similar trend but was not statistically significant (p = 0.069). G. Measurements of ectocranial, mesocranial, and endocranial widths for the sagittal suture with the high dose having significantly increased width on the endocranial edge compared to control (p = 0.019). Similar trends were observed for the ectocranial (p = 0.214) and height dimensions (p = 0.063), but were not statistically significant. All measurements are presented as mean ±SEM. * p = 0.05–0.011; ** p = 0.01–0.001; *** p<0.001.

Analysis of histomorphometry revealed that high dose exposed animals had significantly diminished coronal suture area (more closely approximated osteogenic fronts) compared to control and low dose measures at the 25 day terminal time point p<0.01 (Fig 2D). There does appear to be compensation at the sagittal suture; however, only the low dose had a statistically significant greater area measure than control, p = 0.027 (Fig 2D). Representative suture histology is included as Fig 2A–2C. The histomorphic analysis of a subset of individuals (n = 3 per exposure) was partially corroborated in a larger sample via μCT analysis of suture widths (n = 9 per exposure) with differing measures for both coronal and sagittal suture width by dose (Fig 2E–2G). These analyses were however not significantly different suggesting histology may be a more accurate measure for these variables most likely due to the loss of resolution when using μCT images (13 μm) compared to histological images (7 μm).

### In utero Thyroxine Exposure Drives Increased Proliferation and Osteogenic Expression

**Fig 3:**
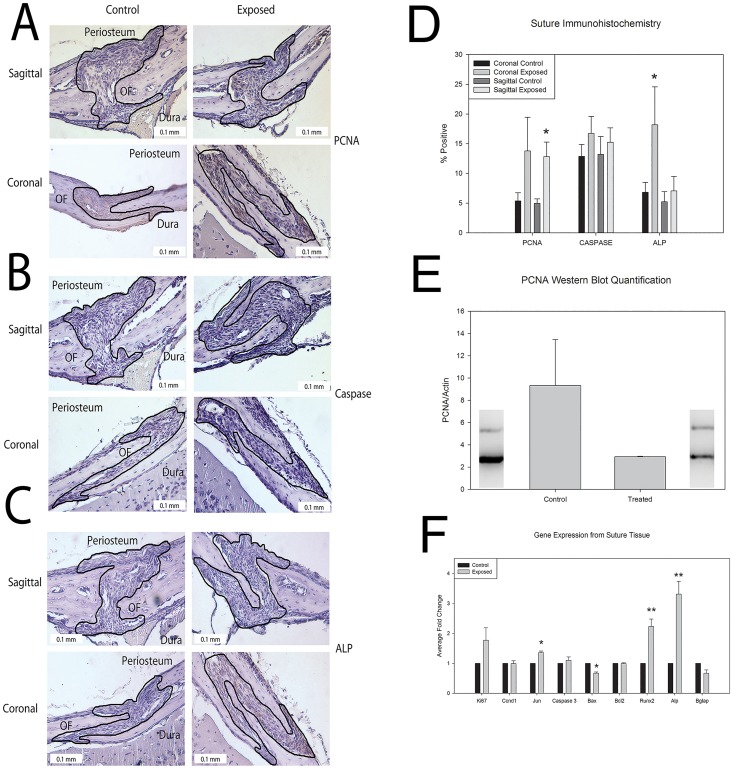
Histological markers of proliferation, bone formation, and apoptosis in control and thyroxine exposed mouse cranial sutures. A-C. Sagittal (top) and coronal (bottom) sutures for control (right) and high dose exposed (left) 20 day post-natal, C57BL6 mice stained for Proliferating Cell Nuclear Antigen (PCNA) (A), Active Caspase 3 (Caspase) (B), and Alkaline Phosphatase (ALP) (C). Note darker staining for PCNA and ALP in the sutures exposed to thyroxine and the relative lack of Caspase staining, regardless of exposure. Sutures are outlined with periosteum above, osteogenic fronts (OF) on either side, and dura below. D. Quantification of the percent positive staining of PCNA, Caspase, and ALP stained coronal and sagittal sutures from control and high dose exposed 20 day post-natal mice confirms significantly more positive staining for PCNA in the sagittal sutures of mice exposed *in utero* to high dose thyroxine as compared to control (p = 0.05). Caspase activity was found to be slightly elevated but not statistically significant in both the coronal (p = 0.068) and sagittal (p = 0.295) sutures. Significantly more positive staining for ALP was found in the coronal sutures of mice exposed *in utero* to high doses of thyroxine as compared to controls (p = 0.024) (n = 3 individuals per exposure x 3 stained sections per suture for each target). E. Western blot analysis of total protein collected from sutures for PCNA suggests increased presence in control samples compared to high dose exposed samples. This was not found to be statistically significant (p = 0.192) F. Gene expression markers of proliferation (*Ki67*, *Ccnd1*, *Jun*), apoptosis (*Caspase 3*, *Bax*, *Bcl2*), and osteogenesis (*Runx2*, *Alp*, *Bglap*) from suture tissue confirm significantly increased expression for a marker of proliferation (*Jun* p = 0.03), significantly decreased expression for a marker apoptosis (*Bax* p = 0.026) and significantly increased expression for markers of osteogenesis (*Runx2* p = 0.023, *Alp* p = 0.01) with exposure to thyroxine *in utero* (n = 3 per exposure). All measurements are presented as mean ±SEM. * p = 0.05–0.011; ** p = 0.01–0.001; *** p<0.001.

As the most significant changes occurred between 20 and 25 days we chose to further investigate 20 day high dose (~667 ng per day) *in utero* exposed sutures histologically for factors driving the recorded morphological changes. Previous research by our group suggests thyroxine targets calvarial cells increasing proliferation and osteogenesis when treated *in vitro* [51]. Western blot analysis of total perisutural tissue PCNA showed increased presence in control samples versus treated, but was not statistically significant, p = 0.1920 (Fig 3E). However, targeted analysis of the isolated suture, via quantified immunohistochemistry shows significantly increased PCNA in the *in utero* thyroxine exposed sagittal sutures compared to controls, p = 0.05 (Fig 3D). No significant difference in active Caspase 3 was found either via western blot (undetected with the exception of the experimental control, S1 Fig) or immunohistochemistry. Slight, but not significant, increases were found in coronal and sagittal sutures with thyroxine exposure (Fig 3D). We did observe a statistically significant increase in alkaline phosphatase (ALP), a marker of bone formation, staining in the coronal sutures of pups exposed *in utero*, p = 0.024 (Fig 3D).

Gene expression data suggest increases with exposure for all markers of proliferation tested, with a statistically significant increase in *Jun* expression, p = 0.03. There was a statistical difference for *Bax*, a marker of apoptosis, p = 0.027. Two osteogenic markers, *Runx2* and *Alp* showed great increase in gene expression in the *in utero* exposed pups (>2 fold increases), both of which were statistically significant (p = 0.023, and p = 0.01 respectively). There was no statistical difference in gene expression for *Bglap*, a marker of mature osteoblasts and osteocytes (Fig 3F).

### In utero Thyroxine Exposure Drives Expression Changes in Htra1/Igf1 at the Cranial Suture

**Fig 4:**
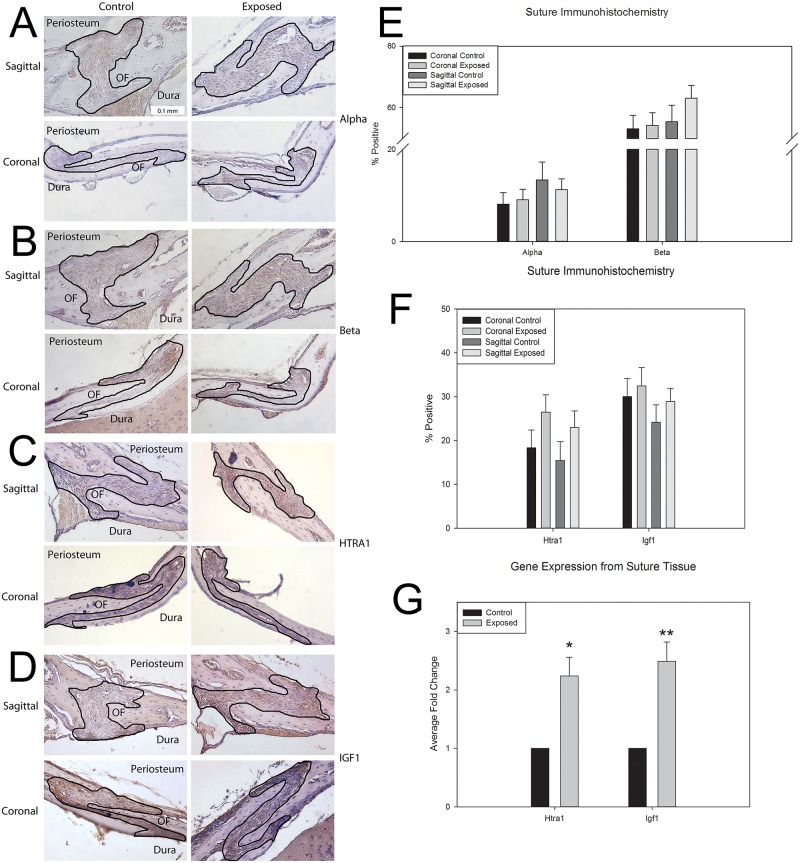
Histological markers for the effect of *in utero* thyroxine exposure on the Htra1/IGF1 signaling axis at the cranial sutures. A-B. Sagittal (top) and coronal (bottom) sutures for control, 20 day post-natal, C57BL6 mice stained for the presence of the alpha (A) and beta (B) forms of the thyroid receptor. Sutures demonstrating the presence of receptors are outlined with periosteum above, osteogenic fronts (OF) on either side, and dura below. C-D. Sagittal (top) and coronal (bottom) sutures from control (right) and high dose exposed (left) 20d post-natal mice stained for HTRA1 (C) and IGF1 (D). Note the greater staining in the sagittal suture from the exposed mice. Sutures are outlined with periosteum above, osteogenic fronts (OF) on either side, and dura below. E-F. Quantification of the percent positive staining of alpha and beta thyroid receptors (E) and HTRA1 and IGF1 (F) stained coronal and sagittal sutures from control and high dose exposed 20 day post-natal mice suggests more positive staining for the presence of HTRA1 in high dose exposed sutures as compared to control. This was not found to be statistically significant (coronal p = 0.166, sagittal p = 0.102) (n = 3 individuals per exposure x 3 stained sections per suture for each target). G. Gene expression for *Htra1* and *Igf1* from suture tissue demonstrate significantly more expression of both targets (p = 0.03 and p = 0.01, respectively) (n = 3 per exposure). All measurements are presented as mean ±SEM. * p = 0.05–0.011; ** p = 0.01–0.001.

Fig 4A and 4B positively demonstrates the presence of alpha and beta forms of the thyroid receptor within the cranial sutures. Interestingly, beta receptor appears to have a greater presence in both the coronal and sagittal suture compared to alpha receptor; however there was no modulation of receptor presence with exposure ((Coronal alpha p = 0.824, beta p = 0.862) (Sagittal alpha p = 0.503, beta p = 0.267)) (Fig 2E). We hypothesized that as *Htra1/Igf1* are targets of thyroid hormone, we would observe modulation of these targets after *in utero* thyroxine exposure. Quantification of immunohistochemistry (Fig 4C and 4D) suggests increased HTRA1 and IGF1 in the coronal and sagittal sutures of the *in utero* exposed animals. However, these increases were not found to be statistically significant (Fig 4F). Gene expression from suture tissue did show significant increases in both *Htra1* (Fold change 2.24+/-0.32, p = 0.03) and *Igf1* (Fold change 2.49 +/- 0.33, p = 0.019) after *in utero* thyroxine exposure (Fig 4G).

### In utero Thyroxine Exposure Downstream Signaling

**Fig 5:**
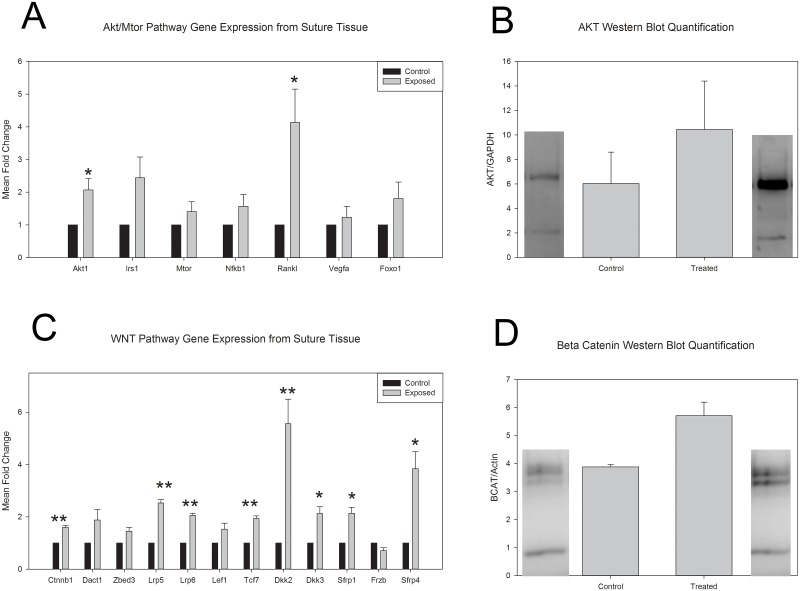
Gene and protein analysis for the effect of *in utero* thyroxine exposure on the Akt and Wnt signaling axis at the cranial sutures. A. Gene expression markers for Akt (*Akt1*), receptor (*Irs1*), and downstream targets (*Mtor*, *Nfkb1*, *Rankl*, *Vegfa*, and *Foxo1*) from suture tissue confirm significantly more expression of *Akt1* (p = 0.05) and *Rankl* (p = 0.03). Several other targets also appeared to have increased expression but were not found to be statistically significant (*Irs1* p = 0.07; *Nfkb1* p = 0.018; *Foxo1* p = 0.16) B. Western blot analysis of protein from total suture tissue for Akt protein suggested increased presence in high dose exposed samples compared to control. This was not found to be statistically significant (p = 0.420) C. Gene expression markers for Wnt co-receptors (*Lrp5*, and *Lrp6*), β-catenin (*Ctnnb1*, *Dact1*, *Zbed3*), downstream targets (*Lef1* and *Tcf7*), agonists (*Dkk2*, *Dkk3*) and antagonists (*Sfrp1*, *Frzb*, and *Sfrp4*). Significant increased expression of the co-receptors Lrp5/6 (p = 0.003 respectively) and β-catenin (p = 0.009) as well as downstream target *Tcf7* (p = 0.005) and agonists *Dkk2/3* (p = 0.009; p = 0.026 receptively) were noted for the thyroxine exposed sutures. Increased expression of agonists due to lack of expression of *Kremen 2* indicate activation of the Wnt pathway, while significant increases in the antagonists *Sfrp1* (p = 0.02) and *Sfrp4* (p = 0.015) indicate either negative feedback on the system or non-canonical function. D. Western blot analysis of protein collected from sutures for β-catenin suggests increased presence of total β-catenin in high dose exposed samples compared to control. This was not found to be statistically significant (p = 0.1660). All measurements are presented as mean ±SEM. * p = 0.05–0.011; ** p = 0.01–0.001.

To investigate potential downstream pathways by which thyroxine exposure causes morphological alterations, gene and protein expression for targets within the Akt and Wnt pathways were evaluated. We found significantly increased gene expression of *Akt1* and *Rankl* (p = 0.05 and p = 0.03 respectively; Fig 5A) with a resulting increase in AKT protein levels in treated animals (Fig 5B). The protein level increase was not found to be statistically significant. Evaluation of protein levels for pAKT indicated levels too low to be detected (S1 Fig). There were statistically significant increases in the gene expression of Wnt co-receptors, *Lrp5* (p = 0.003) and *Lrp6* (p = 0.003), *β-catenin* (p = 0.009), and downstream target *Tcf7* (0.005). However, expression of the antagonists *Sfrp1* (p = 0.02) and *Sfrp4* (p = 0.01) were also found to be significantly increased. Interestingly, *Kremen2* expression was undetectable, indicating that the increased expression of *Dkk2* (p = 0.009) and *Dkk3* (p = 0.026) was most likely to act as an agonist to the *Lrp5/6* co-receptors (Fig 5C). Subsequent protein analysis of total suture tissue β-Catenin showed increased levels after thyroxine treatment (Fig 5D). This difference was not statistically significant.

## Discussion

Overall our data suggest that maternal exogenous thyroxine exposure can drive calvarial growth and suture morphology alterations. This observed alteration is consistent with previously published literature indicating suture narrowing after thyroid treatment in other animal models [52, 53]. We observed the cessation of growth in length of the calvaria consistent with aberrant coronal suture growth. The cessation of growth for the cranial vault length, in conjunction with the compensatory growth of the sagittal suture leading to increased cranial vault width in high dose animals at 25 days, suggests that thyroxine affects growth of the coronal suture. As observed in the representative μCT skull reconstructions, there does appear to be gross craniofacial dysmorphology and aberrant coronal suture morphology after high dose exposure. Our suture histomorphometry and μCT analyses were consistent with these results, with the histomorphometry likely being less error prone due to direct visualization of tissues. The high dose exposed animals had the smallest coronal suture area measures, with low dose exhibiting a milder phenotype. Interestingly, both low and high dose exposed animals exhibited increased area at the sagittal suture, consistent with compensatory growth. As this is a dynamic system, compensatory growth in the sagittal suture may be detected before restriction of the coronal suture can be measured via the available techniques. In fact, low dose exhibited the greatest difference which may indicate growth across the coronal suture would decrease after 25 days for these animals. This could indicate that due to thyroxine being a hormone, the low dose may not be modeling an intermediate effect, but instead we are observing a specific low dose effect. Alternatively, these observations could indicate that the window of susceptibility in the low dose animals has shifted to a later time point. However, as calvarial expansion is almost complete at this age, morphometric differences would not be expected at a later post-natal time point as brain expansion and final vault form is complete by approximately 30 days post-natal [54].

In order to accurately capture the signaling changes that result in the altered morphology of the cranial vault at 25 days, the 20 day time point was used to determine gene and protein alterations within the treated animals. When investigating cellular processes, we concentrated on known effects of thyroid hormone on calvarial cells where increased proliferation and osteogenesis have been observed *in vitro* [51]. Our immunohistochemistry data suggest increased PCNA, a marker of cell proliferation in the sagittal suture, and increased ALP in the coronal suture specifically, consistent with increased bony infiltration and osteoblastic activity at the suture in the high dose exposed animals. The contrasting increase in PCNA levels in control samples in our western blot is most likely due to whole tissue protein being collected allowing for the possibility of the presence hematopoietic cells in the sample which expresses high levels of PCNA, to overshadow minute differences in cell proliferation within the suture itself. Our immunohistochemistry results show a more targeted analysis of the suture, and therefore give a better evaluation of the response of suture cells to thyroxine. This is of great interest as the sagittal suture shows signs of compensatory growth which may be consistent with increased cell proliferation. Our gene expression data corroborates these findings showing elevated expression of proliferation markers. *Jun* exhibited a statistically significant increase after *in utero* exposure to thyroxine. *Jun* has been shown to be important for progression of proliferating cells through G1 phase and to also act as an anti-apoptotic factor [55, 56]. Although *Bax*, a gene related to apoptosis, did demonstrate decreased gene expression, none of the other gene targets were significantly different. In fact, our immunohistochemistry suggests an opposite, although not statistically significant, response of increased active Caspase 3 indicative of more apoptosis. Thus, due to this mixed data, most of which is not statistically significant, it is unlikely excess thyroid hormone disrupts growth via apoptosis of suture cells. Not surprisingly both *Runx2* and *Alp* were observed to have significant gene expression increases in the *in utero* high dose exposed sutures. This is consistent with our immunohistochemistry data and with the literature suggesting thyroid hormones to be osteogenic [26–28, 34]. We do not observe osteocalcin (*Bglap*) to have increased expression. In contrast, it shows a slight decrease in gene expression, although not statistically significant, compared to unexposed controls. This may be the result of active or early osteoblastic activity in the 20 day exposed sutures, as osteocalcin is considered a marker of mature osteoblasts and osteocytes [57].

There was some evidence for alteration in HTRA1/IGF1 signaling axis after *in utero* exposure to thyroxine. It is known that thyroid hormone acts through its receptors to mediate growth in the long bones through modulation of *Htra1/Igf1* signaling [26–28, 34]. Here we demonstrate the presence of both alpha and beta thyroid hormone receptors in the cranial sutures suggesting circulating thyroid hormone can affect the suture through direct mechanisms. While previous studies have shown that expression levels of the alpha receptor were 10 times that of the beta receptor within long bones, we found the presence of the beta receptor is likely higher in the sutures than the alpha receptor. It is possible this result is an artifact of specificity of the antibodies to epitopes on the receptor but is more likely that presence of these receptors is both spatially and temporally regulated independently within different tissues [58–60]. Further, both HTRA1 and IGF1 exhibit elevated presence and significantly increased gene expression in the high dose exposed animals at post-natal day 20. Importantly this is prior to that decrease in growth observed at 25 days post-natal compared to unexposed controls. Overall increased activity of these targets may be responsible for the tissue level changes we observed that lead to craniofacial growth alterations.

To further interrogate the possible signaling cascade activated by thyroxine, targets within the Akt and Wnt pathways were evaluated. Previous studies have shown that thyroid hormone can activate thyroid receptor beta by directly binding to the receptor or indirectly through increased levels of IGF1 [61–63]. Our results seem to support these findings as thyroid receptor beta was shown to be present within both the coronal and sagittal suture areas and gene expression of *Igf1* was significantly elevated with exposure. Binding of the thyroid receptor beta, either directly by T3 or indirectly by IGF1, has been found to activate multiple signaling cascades, specifically the Akt and Wnt pathways[64]. Interestingly, while thyroxine significantly increased expression levels of *Akt1* within the suture tissues, downstream targets of the Akt pathway were not upregulated. This is most likely due to the fact that Akt can directly phosphorylate β-Catenin promoting its nuclear translocation and subsequent increased transcriptional activity [62, 65] in a cascade that more closely mimics the Wnt pathway. Thyroid receptor beta activation has also been found to facilitate Akt translocation to the nucleus in a phosphoinositide 3-kinase dependent manner [61, 63, 66] without affecting total Akt [67], which could explain that while thyroxine treatment showed significantly increased gene expression, the observed increased protein presence of Akt was not significant.

To evaluate if thyroxine activated Wnt signaling, targets within the pathway were assessed for gene regulation. Our results showed significant upregulation of several targets within the pathway including the Wnt co-receptors, *Lrp5* and *Lrp6*, agonists, *Dkk2* and *Dkk3*, *β-catenin* and downstream target, *Tcf7*. Subsequent increase in β-Catenin protein levels was also observed. While these results seem to indicate a Wnt pathway signaling cascade is activated by thyroxine exposure, the significant increased expression of the Wnt antagonists, *Sfrp1* and *Sfrp4*, might indicate that this effect stimulates a negative feedback loop or increases in non-canonical signaling. Alternatively, β-catenin is also known to translocate to the nucleus following phosphorylation by Akt independent of canonical Wnt signaling [65], which may indicate cross-talk between the Akt and Wnt pathways that circumvents any negative feedback from *Sfrp1* and *Sfrp4*. Future *in vitro* cell studies evaluating Akt and β-Catenin interaction and more specifically their translocation into the nucleus, are necessary to characterize the nuances of the signaling cascade activated by exogenous thyroxine exposure.

Overall it appears exogenous thyroid hormone exposures may be linked to calvarial growth and suture morphology alterations. This seems to involve increased activity of the Htra1/Igf1, Akt, and Wnt signaling axes leading to proliferation and osteoblastic activity at the calvarial sutures at 20 days post-natal. Increased activity of this nature disrupts homeostasis of the sutures which are normally a fibroblast rich, undifferentiated environment that is permissive of brain expansion. Recently, cells within the suture have been identified as plastic or to be “mesenchymal stem cells” [68, 69]. These resident cell populations may play an integral role in maintenance of the suture by either cell/cell signaling or the mere presence of cells that are not osteoblasts within the suture environment. Although our data suggest increased proliferation and osteogenesis as a key to exogenous thyroid hormone driven calvarial growth alteration and disruption, the susceptible resident cell populations remain to be interrogated. While previous research has indicated crosstalk between the Htra1/Igf1, Akt, and Wnt pathways after thyroid exposure leading to a synergistic effect [63], further characterization of the signaling cascade is required to determine how thyroid exposure results in craniosynostosis. Future research should concentrate on the culprit cell type(s) responsive and responsible for this caustic interaction with a goal of developing a cell based targeted therapy to decrease incidence of craniosynostosis caused by exogenous thyroid hormone exposure.

## Supporting Information

S1 Fig Western blot analysis of Caspase 3 and pAKT. A Western blot analysis of Caspase 3 positive and negative controls and control or thryoxine exposed sutures. B. Western blot analysis of pAKT and control or thryoxine exposed sutures.
(TIF)

S1 Table Quantitative qRT-PCR TaqMan Assay (Applied Biosystems). List of all primers used within this study.
(DOCX)

## References

[1] Richardson S, Browne ML, Rasmussen SA, Druschel CM, Sun L, Jabs EW, et al Associations between periconceptional alcohol consumption and craniosynostosis, omphalocele, and gastroschisis. Birth defects research Part A, Clinical and molecular teratology. 2011;91(7):623–30. 10.1002/bdra.20823 21630421 PMC6042859

[2] Browne ML, Hoyt AT, Feldkamp ML, Rasmussen SA, Marshall EG, Druschel CM, et al Maternal caffeine intake and risk of selected birth defects in the National Birth Defects Prevention Study. Birth defects research Part A, Clinical and molecular teratology. 2011;91(2):93–101. 10.1002/bdra.20752 21254365 PMC6042844

[3] Reefhuis J, Honein MA, Schieve LA, Rasmussen SA, National Birth Defects Prevention S. Use of clomiphene citrate and birth defects, National Birth Defects Prevention Study, 1997–2005. Human reproduction. 2011;26(2):451–7. 10.1093/humrep/deq313 21112952

[4] Carmichael SL, Rasmussen SA, Lammer EJ, Ma C, Shaw GM, National Birth Defects Prevention S. Craniosynostosis and nutrient intake during pregnancy. Birth defects research Part A, Clinical and molecular teratology. 2010;88(12):1032–9. 10.1002/bdra.20717 20842649 PMC3136510

[5] Rasmussen SA, Yazdy MM, Carmichael SL, Jamieson DJ, Canfield MA, Honein MA. Maternal thyroid disease as a risk factor for craniosynostosis. Obstetrics and gynecology. 2007;110(2 Pt 1):369–77. 10.1097/01.AOG.0000270157.88896.76 17666613

[6] Twigg SR, Wilkie AO. A Genetic-Pathophysiological Framework for Craniosynostosis. American journal of human genetics. 2015;97(3):359–77. Epub 2015/09/05. 10.1016/j.ajhg.2015.07.006 26340332 PMC4564941

[7] Heuze Y, Holmes G, Peter I, Richtsmeier JT, Jabs EW. Closing the Gap: Genetic and Genomic Continuum from Syndromic to Nonsyndromic Craniosynostoses. Current genetic medicine reports. 2014;2(3):135–45. Epub 2015/07/07. 10.1007/s40142-014-0042-x 26146596 PMC4489147

[8] Twigg SR, Wilkie AO. New insights into craniofacial malformations. Hum Mol Genet. 2015;24(R1):R50–9. 10.1093/hmg/ddv228 26085576 PMC4571997

[9] Carmichael S, Ma C, Rasmussen S, Cunningham M, Browne M, Dosiou C, et al Craniosynostosis and risk factors related to thyroid dysfunction. American journal of medical genetics Part A. 2015. 10.1002/ajmg.a.36953 PMC4768483 25655789

[10] Ardalan M, Rafati A, Nejat F, Farazmand B, Majed M, El Khashab M. Risk factors associated with craniosynostosis: a case control study. Pediatric neurosurgery. 2012;48(3):152–6. 10.1159/000346261 23428561

[11] Hashmi SS, Canfield MA, Marengo L, Moffitt KB, Belmont JW, Freedenberg D, et al The association between neonatal thyroxine and craniosynostosis, Texas, 2004–2007. Birth defects research Part A, Clinical and molecular teratology. 2012;94(12):1004–9. 10.1002/bdra.23077 23109112

[12] McNab T, Ginsberg J. Use of anti-thyroid drugs in euthyroid pregnant women with previous Graves' disease. Clinical and investigative medicine Medecine clinique et experimentale. 2005;28(3):127–31. 16021986

[13] Radetti G, Zavallone A, Gentili L, Beck-Peccoz P, Bona G. Foetal and neonatal thyroid disorders. Minerva pediatrica. 2002;54(5):383–400. 12244277

[14] Zimmerman D. Fetal and neonatal hyperthyroidism. Thyroid: official journal of the American Thyroid Association. 1999;9(7):727–33. 10447021 10.1089/thy.1999.9.727

[15] Krude H, Biebermann H, Krohn HP, Dralle H, Gruters A. Congenital hyperthyroidism. Experimental and clinical endocrinology & diabetes: official journal, German Society of Endocrinology [and] German Diabetes Association. 1997;105 Suppl 4:6–11. 10.1055/s-0029-1211924 9439907

[16] Johnston MC, Bronsky PT. Prenatal craniofacial development: new insights on normal and abnormal mechanisms. Critical reviews in oral biology and medicine: an official publication of the American Association of Oral Biologists. 1995;6(4):368–422. 10.1177/10454411950060040601 8664424

[17] Burr W. Prescribing in pregnancy. Thyroid disease. Clinics in obstetrics and gynaecology. 1986;13(2):277–90. 2426028

[18] Penfold JL, Simpson DA. Premature craniosynostosis-a complication of thyroid replacement therapy. The Journal of pediatrics. 1975;86(3):360–3. 1113223 10.1016/s0022-3476(75)80963-2

[19] Jin SW, Sim KB, Kim SD. Development and Growth of the Normal Cranial Vault: An Embryologic Review. Journal of Korean Neurosurgical Society. 2016;59(3):192–6. Epub 2016/05/27. 10.3340/jkns.2016.59.3.192 27226848 PMC4877539

[20] Opperman LA. Cranial sutures as intramembranous bone growth sites. Developmental dynamics: an official publication of the American Association of Anatomists. 2000;219(4):472–85. Epub 2000/11/21. 11084647 10.1002/1097-0177(2000)9999:9999<::AID-DVDY1073>3.0.CO;2-F

[21] Chadduck WM, Chadduck JB, Boop FA. The subarachnoid spaces in craniosynostosis. Neurosurgery. 1992;30(6):867–71. 1614588 10.1227/00006123-199206000-00008

[22] Arnaud E, Renier D, Marchac D. Prognosis for mental function in scaphocephaly. J Neurosurg. 1995;83(3):476–9. 10.3171/jns.1995.83.3.0476 7666225

[23] Miller MT. Ocular findings in craniosynostosis In: Cohen MM Jr., MacLean RE, editors. Craniosynostosis: Diagnosis, Evaluation, and Management. New York: Oxford University Press; 2000 p. 184–96.

[24] McNabb FMA. Thyroid hormones. Englewood Cliffs, N.J.: Prentice Hall; 1992 xv, 283 p. p.

[25] Oppenheimer JH, Samuels HH, Apriletti JW. Molecular basis of thyroid hormone action. New York: Academic Press; 1983 xv, 498 p. p.

[26] Kim HY, Mohan S. Role and Mechanisms of Actions of Thyroid Hormone on the Skeletal Development. Bone Res. 2013;1(2):146–61. 10.4248/BR201302004 26273499 PMC4472099

[27] Bassett JH, Williams GR. Role of thyroid hormones in skeletal development and bone maintenance. Endocr Rev. 2016:er20151106. 10.1210/er.2015-1106 PMC4823381 26862888

[28] Williams GR. Thyroid hormone actions in cartilage and bone. Eur Thyroid J. 2013;2(1):3–13. 10.1159/000345548 24783033 PMC3821494

[29] Glanz S, Mirsaidi A, Lopez-Fagundo C, Filliat G, Tiaden AN, Richards PJ. Loss-of-Function of HtrA1 Abrogates All-Trans Retinoic Acid-Induced Osteogenic Differentiation of Mouse Adipose-Derived Stromal Cells Through Deficiencies in p70S6K Activation. Stem Cells Dev. 2016;25(9):687–98. 10.1089/scd.2015.0368 26950191

[30] Robubi A, Berger C, Schmid M, Huber KR, Engel A, Krugluger W. Gene expression profiles induced by growth factors in in vitro cultured osteoblasts. Bone Joint Res. 2014;3(7):236–40. 10.1302/2046-3758.37.2000231 25057185 PMC4112778

[31] Bu YH, He YL, Zhou HD, Liu W, Peng D, Tang AG, et al Insulin receptor substrate 1 regulates the cellular differentiation and the matrix metallopeptidase expression of preosteoblastic cells. J Endocrinol. 2010;206(3):271–7. 10.1677/JOE-10-0064 20525764

[32] Ulici V, Hoenselaar KD, Gillespie JR, Beier F. The PI3K pathway regulates endochondral bone growth through control of hypertrophic chondrocyte differentiation. BMC Dev Biol. 2008;8:40 10.1186/1471-213X-8-40 18405384 PMC2329617

[33] Pirinen S. Endocrine regulation of craniofacial growth. Acta odontologica Scandinavica. 1995;53(3):179–85. 7572094 10.3109/00016359509005969

[34] Wojcicka A, Bassett JH, Williams GR. Mechanisms of action of thyroid hormones in the skeleton. Biochim Biophys Acta. 2013;1830(7):3979–86. 10.1016/j.bbagen.2012.05.005 22634735

[35] Glinoer D. Thyroid hyperfunction during pregnancy. Thyroid: official journal of the American Thyroid Association. 1998;8(9):859–64. 9777758 10.1089/thy.1998.8.859

[36] Rasmussen SA, Yazdy MM, Frias JL, Honein MA. Priorities for public health research on craniosynostosis: summary and recommendations from a Centers for Disease Control and Prevention-sponsored meeting. American journal of medical genetics Part A. 2008;146A(2):149–58. 10.1002/ajmg.a.32106 18080327

[37] Krause K, Weiner J, Hones S, Kloting N, Rijntjes E, Heiker JT, et al The Effects of Thyroid Hormones on Gene Expression of Acyl-Coenzyme A Thioesterases in Adipose Tissue and Liver of Mice. Eur Thyroid J. 2015;4(Suppl 1):59–66. 10.1159/000437304 26601074 PMC4640296

[38] Capuco AV, Kahl S, Jack LJ, Bishop JO, Wallace H. Prolactin and growth hormone stimulation of lactation in mice requires thyroid hormones. Proc Soc Exp Biol Med. 1999;221(4):345–51. 10460696 10.1046/j.1525-1373.1999.d01-91.x

[39] Thordarson G, Fielder P, Lee C, Hom YK, Robleto D, Ogren L, et al Mammary gland differentiation in hypophysectomized, pregnant mice treated with corticosterone and thyroxine. Biol Reprod. 1992;47(4):676–82. 1382632 10.1095/biolreprod47.4.676

[40] Capelo LP, Beber EH, Huang SA, Zorn TM, Bianco AC, Gouveia CH. Deiodinase-mediated thyroid hormone inactivation minimizes thyroid hormone signaling in the early development of fetal skeleton. Bone. 2008;43(5):921–30. Epub 2008/08/07. 10.1016/j.bone.2008.06.020 18682303 PMC4683160

[41] Darnerud PO, Morse D, Klasson-Wehler E, Brouwer A. Binding of a 3,3', 4,4'-tetrachlorobiphenyl (CB-77) metabolite to fetal transthyretin and effects on fetal thyroid hormone levels in mice. Toxicology. 1996;106(1–3):105–14. Epub 1996/01/08. 8571380 10.1016/0300-483x(95)03169-g

[42] Dulhunty AF, Gage PW, Lamb GD. Differential effects of thyroid hormone on T-tubules and terminal cisternae in rat muscles: an electrophysiological and morphometric analysis. Journal of muscle research and cell motility. 1986;7(3):225–36. Epub 1986/06/01. 3734053 10.1007/BF01753555

[43] Juriloff DM, Harris MJ. Thyroxine-induced differential mortality of cleft lip mouse embryos: dose- and time-response studies of the A/WySn strain. Teratology. 1985;31(3):319–29. Epub 1985/06/01. 10.1002/tera.1420310302 4040275

[44] Lamb JCt, Harris MW, McKinney JD, Birnbaum LS. Effects of thyroid hormones on the induction of cleft palate by 2,3,7,8-tetrachlorodibenzo-p-dioxin (TCDD) in C57BL/6N mice. Toxicology and applied pharmacology. 1986;84(1):115–24. Epub 1986/06/15. 3715858 10.1016/0041-008x(86)90420-5

[45] Lamberg BA, Helenius T, Liewendahl K. Assessment of thyroxine suppression in thyroid carcinoma patients with a sensitive immunoradiometric TSH assay. Clinical endocrinology. 1986;25(3):259–63. Epub 1986/09/01. 3791667 10.1111/j.1365-2265.1986.tb01690.x

[46] Kilkenny C, Browne WJ, Cuthill IC, Emerson M, Altman DG. Improving bioscience research reporting: the ARRIVE guidelines for reporting animal research. PLoS Biol. 2010;8(6):e1000412 10.1371/journal.pbio.1000412 20613859 PMC2893951

[47] Parsons TE, Weinberg SM, Khaksarfard K, Howie RN, Elsalanty M, Yu JC, et al Craniofacial shape variation in Twist1+/- mutant mice. Anatomical record. 2014;297(5):826–33. 10.1002/ar.22899 24585549

[48] Proff P, Weingartner J, Bayerlein T, Reicheneder C, Fanghanel J, Bill J. Histological and histomorphometric study of growth-related changes of cranial sutures in the animal model. J Craniomaxillofac Surg. 2006;34 Suppl 2:96–100. 17071401 10.1016/S1010-5182(06)60021-8

[49] Varghese F, Bukhari AB, Malhotra R, De A. IHC Profiler: an open source plugin for the quantitative evaluation and automated scoring of immunohistochemistry images of human tissue samples. PLoS One. 2014;9(5):e96801 10.1371/journal.pone.0096801 24802416 PMC4011881

[50] Yuan JS, Reed A, Chen F, Stewart CN Jr. Statistical analysis of real-time PCR data. BMC bioinformatics. 2006;7:85 10.1186/1471-2105-7-85 16504059 PMC1395339

[51] Cray JJ Jr., Khaksarfard K, Weinberg SM, Elsalanty M, Yu JC. Effects of thyroxine exposure on osteogenesis in mouse calvarial pre-osteoblasts. PLoS One. 2013;8(7):e69067 Epub 2013/08/13. 10.1371/journal.pone.0069067 23935926 PMC3720861

[52] Akita S, Hirano A, Fujii T. Identification of IGF-I in the calvarial suture of young rats: histochemical analysis of the cranial sagittal sutures in a hyperthyroid rat model. Plastic and reconstructive surgery. 1996;97(1):1–12. Epub 1996/01/01. 8532765 10.1097/00006534-199601000-00001

[53] Akita S, Nakamura T, Hirano A, Fujii T, Yamashita S. Thyroid hormone action on rat calvarial sutures. Thyroid: official journal of the American Thyroid Association. 1994;4(1):99–106. Epub 1994/01/01. 8054867 10.1089/thy.1994.4.99

[54] Vecchione L, Miller J, Byron C, Cooper GM, Barbano T, Cray J, et al Age-related changes in craniofacial morphology in GDF-8 (myostatin)-deficient mice. Anatomical record. 2010;293(1):32–41. Epub 2009/11/10. 10.1002/ar.21024 PMC3113544 19899116

[55] Wisdom R, Johnson RS, Moore C. c-Jun regulates cell cycle progression and apoptosis by distinct mechanisms. EMBO J. 1999;18(1):188–97. 10.1093/emboj/18.1.188 9878062 PMC1171114

[56] Schreiber M, Kolbus A, Piu F, Szabowski A, Mohle-Steinlein U, Tian J, et al Control of cell cycle progression by c-Jun is p53 dependent. Genes Dev. 1999;13(5):607–19. 10072388 10.1101/gad.13.5.607 PMC316508

[57] Long F. Building strong bones: molecular regulation of the osteoblast lineage. Nat Rev Mol Cell Biol. 2012;13(1):27–38. 10.1038/nrm3254 22189423

[58] Murphy E, Williams GR. The thyroid and the skeleton. Clinical endocrinology. 2004;61(3):285–98. Epub 2004/09/10. 10.1111/j.1365-2265.2004.02053.x 15355444

[59] O'Shea PJ, Harvey CB, Suzuki H, Kaneshige M, Kaneshige K, Cheng SY, et al A thyrotoxic skeletal phenotype of advanced bone formation in mice with resistance to thyroid hormone. Molecular endocrinology (Baltimore, Md). 2003;17(7):1410–24. Epub 2003/04/05. 10.1210/me.2002-0296 12677005

[60] Stevens DA, Harvey CB, Scott AJ, O'Shea PJ, Barnard JC, Williams AJ, et al Thyroid hormone activates fibroblast growth factor receptor-1 in bone. Molecular endocrinology (Baltimore, Md). 2003;17(9):1751–66. Epub 2003/06/14. 10.1210/me.2003-0137 12805413

[61] Borgatti P, Martelli AM, Bellacosa A, Casto R, Massari L, Capitani S, et al Translocation of Akt/PKB to the nucleus of osteoblast-like MC3T3-E1 cells exposed to proliferative growth factors. FEBS letters. 2000;477(1–2):27–32. Epub 2000/07/19. 10899305 10.1016/s0014-5793(00)01758-0

[62] Fang D, Hawke D, Zheng Y, Xia Y, Meisenhelder J, Nika H, et al Phosphorylation of beta-catenin by AKT promotes beta-catenin transcriptional activity. The Journal of biological chemistry. 2007;282(15):11221–9. Epub 2007/02/09. 10.1074/jbc.M611871200 17287208 PMC1850976

[63] Wang L, Shao YY, Ballock RT. Thyroid hormone-mediated growth and differentiation of growth plate chondrocytes involves IGF-1 modulation of beta-catenin signaling. Journal of bone and mineral research: the official journal of the American Society for Bone and Mineral Research. 2010;25(5):1138–46. Epub 2010/03/05. 10.1002/jbmr.5 PMC3123724 20200966

[64] Bassett JH, Williams GR. Role of Thyroid Hormones in Skeletal Development and Bone Maintenance. Endocrine reviews. 2016;37(2):135–87. Epub 2016/02/11. 10.1210/er.2015-1106 26862888 PMC4823381

[65] Rybchyn MS, Slater M, Conigrave AD, Mason RS. An Akt-dependent increase in canonical Wnt signaling and a decrease in sclerostin protein levels are involved in strontium ranelate-induced osteogenic effects in human osteoblasts. The Journal of biological chemistry. 2011;286(27):23771–9. Epub 2011/05/14. 10.1074/jbc.M111.251116 21566129 PMC3129158

[66] Gauthier K, Flamant F. Nongenomic, TRbeta-dependent, thyroid hormone response gets genetic support. Endocrinology. 2014;155(9):3206–9. Epub 2014/08/26. 10.1210/en.2014-1597 25152174

[67] Cao X, Kambe F, Moeller LC, Refetoff S, Seo H. Thyroid hormone induces rapid activation of Akt/protein kinase B-mammalian target of rapamycin-p70S6K cascade through phosphatidylinositol 3-kinase in human fibroblasts. Molecular endocrinology (Baltimore, Md). 2005;19(1):102–12. Epub 2004/09/25. 10.1210/me.2004-0093 15388791

[68] Maruyama T, Jeong J, Sheu TJ, Hsu W. Stem cells of the suture mesenchyme in craniofacial bone development, repair and regeneration. Nature communications. 2016;7:10526 Epub 2016/02/03. 10.1038/ncomms10526 26830436 PMC4740445

[69] Zhao H, Feng J, Ho TV, Grimes W, Urata M, Chai Y. The suture provides a niche for mesenchymal stem cells of craniofacial bones. Nature cell biology. 2015;17(4):386–96. Epub 2015/03/24. 10.1038/ncb3139 25799059 PMC4380556

