# Supplementary figures and images for: Effects of In Utero Thyroxine Exposure on Murine Cranial Suture Growth
Source: PLoS One. 2016 Dec 13;11(12):e0167805. doi: 10.1371/journal.pone.0167805 (PMC5154521; doi:10.1371/journal.pone.0167805)

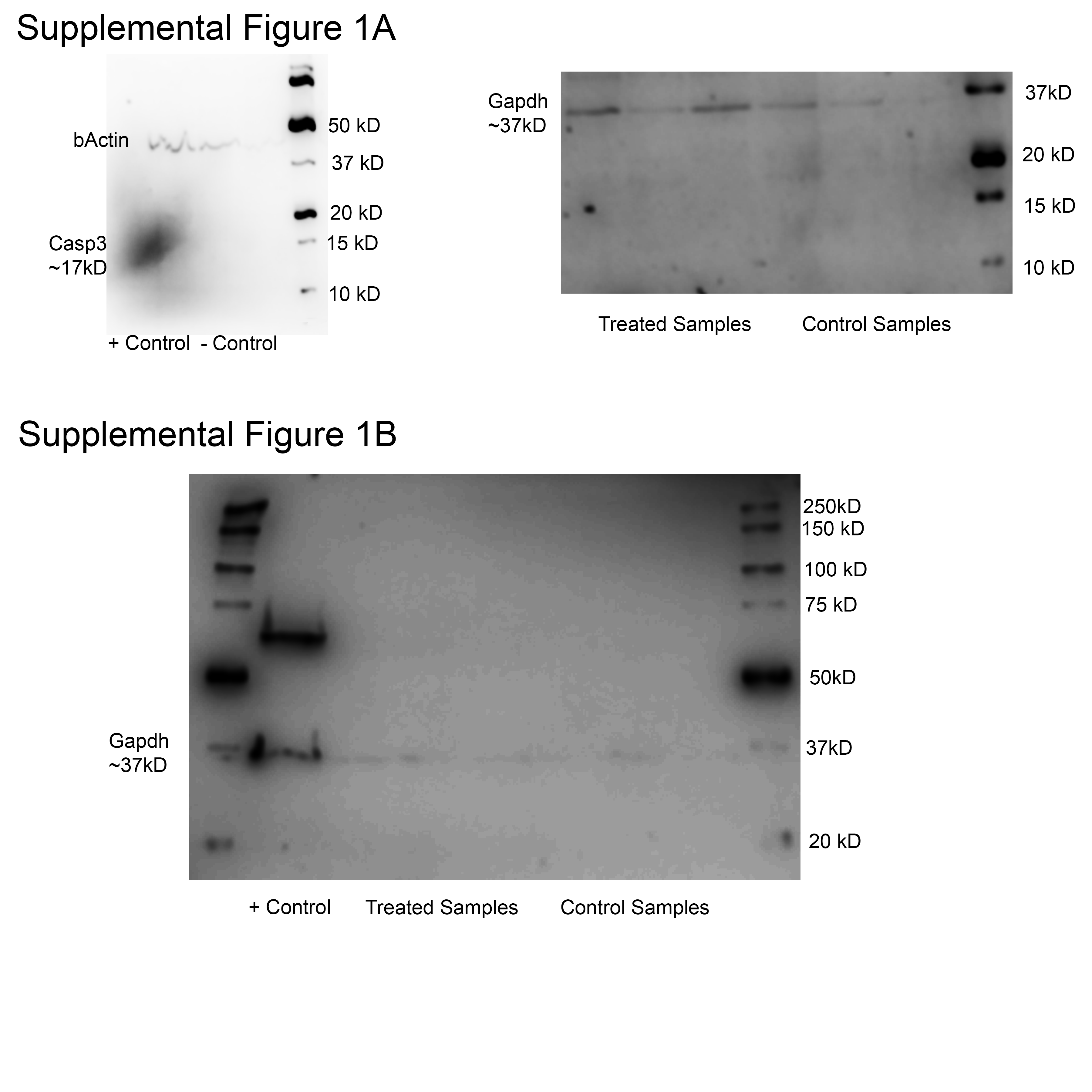

Supplement: S1 Fig — A Western blot analysis of Caspase 3 positive and negative controls and control or thryoxine exposed sutures. B. Western blot analysis of pAKT and control or thryoxine exposed sutures. (TIF) [file pone.0167805.s001.tif]
