# Supplementary material for: Effects of In Utero Thyroxine Exposure on Murine Cranial Suture Growth
Source: PLoS One. 2016 Dec 13;11(12):e0167805. doi: 10.1371/journal.pone.0167805 (PMC5154521; doi:10.1371/journal.pone.0167805)
Supplement: S1 Table — List of all primers used within this study. (DOCX) [file pone.0167805.s002.docx]

**Table 1: Quantitative qRT-PCR TaqMan Assay (Applied Biosystems)**

| *Gene Symbol* | *Gene Name* | *Assay ID* |
| --- | --- | --- |
| Htra1 | HtrA Serine Peptidase 1 | Mm00479892_m1 |
| Igf1 | Insulin Like Growth Factor 1 | Mm00439560_m1 |
| Akt1 | Thymoma Viral Proto-Oncogene 1 | Mm01331626_m1 |
| Irs1 | Insulin Receptor Substrate 1 | Mm01278327_m1 |
| Mtor | Mechanistic Target of Rapamycin (Serine/Threonine Kinase) | Mm00444968_m1 |
| Nfkb1 | Nuclear Factor of Kappa Light Polypeptide Gene Enhancer in B Cells 1 | Mm00476361_m1 |
| Rankl | Tumor Necrosis Factor (ligand) Superfamily, Member 11 | Mm00441906_m1 |
| Vegfa | Vascular Endothelial Growth Factor A | Mm00437306_m1 |
| Foxo1 | Forkhead Box O1 | Mm00490671_m1 |
| Ctnnb1 | Catenin (Cadherin Associated Protein), beta 1 | Mm00483039_m1 |
| Dact1 | Dapper Homolog 1, Antagonist of Beta Catenin | Mm00458117_m1 |
| Zbed3 | Zinc Finger, BED Domain Containing 3 | Mm00511271_m1 |
| Lrp5 | Low Density Lipoprotein Receptor-Related Protein 5 | Mm01227476_m1 |
| Lrp6 | Low Density Lipoprotein Receptor-Related Protein 6 | Mm00999795_m1 |
| Lef1 | Lymphoid Enhancer Binding Factor 1 | Mm00550265_m1 |
| Tcf7 | Transcription Factor 7, T Cell Specific | Mm00493445_m1 |
| Dkk2 | Dickkopf Homolog 2 | Mm01322146_m1 |
| Dkk3 | Dickkopf Homolog 3 | Mm00443800_m1 |
| Sfrp1 | Secreted Frizzled-Related Protein 1 | Mm00489161_m1 |
| Frzb | Frizzled Related Protein (*Sfrp3*) | Mm00441378_m1 |
| Sfrp4 | Secreted Frizzled-Related Protein 4 | Mm00840104_m1 |
| Ki67 | Marker of Proliferation Ki67 | Mm01278617_m1 |
| Ccnd1 | Cyclin D1 | Mm00432359_m1 |
| Jun | Jun Proto-Oncogene | Mm00495062_s1 |
| Casp3 | Caspase 3 | Mm01195085_m1 |
| Bax | BCL Associated X Protein | Mm0043205_m1 |
| Bcl2 | B-Cell CLL/Lymphoma 2 | Mm00477631_m1 |
| Runx2 | Runt Related Transcription Factor 2 | Mm00501584_m1 |
| Alp | Alkaline Phosphatase | Mm00475834_m1 |
| Bglap | Bone Gamma-Carboxyglutamate Protein 3 | Mm01741771_g1 |
| 18S | 18S ribosomal RNA | Mm03928990_g1 |
